# Stocking African catfish in Lake Victoria provides effective biocontrol of snail vectors of *Schistosoma mansoni*

**DOI:** 10.1371/journal.pntd.0013490

**Published:** 2025-09-03

**Authors:** Roland Proud, Fiona Allan, Andrew Whiston, Robert Kayanda, Safari Kinung’hi, Teckla Angelo, Yasinta D. Sylivester, Hillary D. J. Mrosso, Benedicto B. Kashindye, Mboni Elison, Martin J. Cox, Yang Yang, Andrew Chamberlin, Ian L. Boyd, David J. Civitello, Giulio A. De Leo, Andrew S. Brierley

**Affiliations:** 1 Pelagic Ecology Research Group, School of Biology, Gatty Marine Laboratory, Scottish Oceans Institute, University of St Andrews, St Andrews, Fife, United Kingdom; 2 Cupar Analytics Ltd, Cupar, United Kingdom; 3 RAS Technology Ltd, St Andrews, United Kingdom; 4 Lake Victoria Fisheries Organization, Jinja, Uganda; 5 National Institute for Medical Research (NIMR), Mwanza Centre, Mwanza, Tanzania; 6 Nelson Mandela African Institution of Science and Technology, Arusha, Tanzania; 7 Tanzania Fisheries Research Institute (TAFIRI), Mwanza Centre, Mwanza, Tanzania; 8 Integrated Digital East Antarctica, Australian Antarctic Division, Kingston, Australia; 9 The Lyell Centre, Heriot-Watt University, Research Avenue South, Edinburgh, United Kingdom; 10 Department of Oceans and of Earth System Science, Stanford University, California, United States of America; 11 Department of Biology, Emory University, Atlanta, GeorgiaUnited States of America; Sun Yat-Sen University, CHINA

## Abstract

In areas of high infection prevalence, effective control of schistosomiasis – one of the most important Neglected Tropical Diseases – requires supplementing medical treatment with interventions targeted at the environmental reservoir of disease. In addition to provision of clean water, reliable sanitation, and molluscicide use to control the obligate intermediate host snail, top-down biological control of parasite-competent snails has recently gained increasing interest in the scientific community. However, evidence that natural predators can effectively reduce snail abundance and, ultimately, transmission risk to vulnerable human populations remains limited. In this study, we used a Before-After-Control-Intervention (BACI) design implemented in seven lakeside areas, including three intervention areas and four control areas, on the southern shores of Lake Victoria (Tanzania) in 2019–2023. We tested whether the restoration of African catfish, *Clarias gariepinus*, a native species of commercial value, could reduce both the abundance of *Biomphalaria* snails (intermediate hosts of *Schistosoma mansoni*) and infection intensity in school age children (SAC). Where catfish were restored, mean site-level snail counts declined by 57% (95% CI: 29.4%, 74.3%). At primary schools located within each area, SAC infection intensity (mean parasite egg count in stool samples) also decreased significantly by 55% (95% CI: 26%, 73%). This study shows that natural predators of host snails have the potential for schistosomiasis control. Scaling up to a lake-wide approach will require systemic intervention, with snail host control contributing to a broader framework for schistosomiasis management.

## Author summary

*Schistosoma mansoni* is a water-borne parasite of humans that requires freshwater snail vectors (i.e., snails capable of carrying and transmitting disease) to complete its life cycle. More than 250 million people require preventive treatment for schistosomiasis and about 800 million are at risk of infection. In hyperendemic regions, effective control of schistosomiasis is required to complement medical treatment with interventions aimed at reducing snail vector abundance. In this study we tested whether the restoration of the African catfish, a native species of both commercial and nutritional value that has been largely overfished in Lake Victoria, Eastern Africa, can be an effective agent of biological control against schistosome-competent snails. We found that the restoration of the African catfish in the transmission sites in seven lake areas in Tanzania reduced snail vector abundance and transmission risk in the human population. Our study provides supportive evidence that natural enemies of snails can serve as biological control agents against schistosome-parasite snail vectors. In combination with preventive chemotherapy, biocontrol of snail vectors using catfish can help reduce disease burden in lakeside communities, while also offering opportunities for local fishers to provide food locally or for export.

## Introduction

Lake Victoria in East Africa is the world’s largest tropical freshwater lake and has a rapidly expanding human lakeside population [1]. Most of the population in this region live in poverty without access to safe water or effective sanitation [2]. Life and economy are bound tightly to the lake, which provides drinking water, fishing opportunities and washing, bathing and toilet sites [2,3]. Daily water contact activities expose the lakeside population to the waterborne parasite *Schistosoma mansoni*, the causative agent of intestinal schistosomiasis [4,5]. Transmission occurs because the obligate intermediate host of the parasites, i.e., snails of the genus *Biomphalaria* (hereafter ‘snail vectors’), thrive in nearshore lake environments [5,6]. It has been hypothesized that reducing snail abundance through biological control might be an effective way to control schistosomiasis transmission risk [7–9]. The African catfish, *Clarias gariepinus*, a Lake Victoria endemic species and a voracious predator of the snail vector [10], is in decline within the lake [11]. It is possible that this decline promotes persistent high levels of infection within lakeside communities (up to 100%), despite the increased level of preventive chemotherapy over the past decade [12]. This has raised the question: can the restoration of catfish populations at the transmission sites control schistosomiasis transmission risk by reducing the abundance of snail vectors?

Schistosomiasis is a significant source of morbidity and mortality, in particular in sub-Saharan Africa [13]. Depending on the species of schistosome, the parasite can infect the mesenteric and pelvic veins resulting in various pathologies associated with intestinal, hepatosplenic and urogenital disease [14]. Here, we focus on *S. mansoni* which is transmitted within the lake and bordering lagoon/marsh waters. A particular aspect of morbidity arises from the high prevalence of infection in school aged children (SAC), which can result in stunted growth, anaemia [15] and many related issues concerned with child development. Schistosomiasis can be treated with anti-helminthics; the main drug used for chemotherapy is praziquantel (PZQ), which has been used with success to reduce morbidity and prevalence [16–19]. The World Health Organization (WHO) recommends regular treatment (dependent on infection prevalence in a region) of SAC with PZQ as a way of effectively reducing disease prevalence and morbidity [20].

Campaigns to control schistosomiasis using PZQ have reduced disease prevalence and resulting morbidity. These programmes, however, can suffer from high implementation costs and limited effectiveness because PZQ administration does not prevent reinfection following treatment. One study estimated that to control schistosomiasis among SAC in sub-Saharan Africa would require 123 million doses of PZQ annually [21]. PZQ only works on adult schistosome worms so infections with immature worms can persist and would require follow up treatments to fully clear the infection. Treatment strategies are challenged by relatively low investment in drug discovery and development, logistical issues such as drug distribution: a particular problem during the pandemic and in politically unstable countries. The WHO guidelines [22] highlight the need for further integrated control strategies to meet ambitious 2030 targets, such as “Recommendation 6”, which calls for a verification framework that incorporates testing for infection in humans, snails and non-human mammalian hosts. So long as there is a significant environmental reservoir of the parasites causing schistosomiasis, medical treatment in the form of preventive chemotherapy is unlikely to lead to a viable control strategy for schistosomiasis.

*S. mansoni* within the human populations is endemic in most of Eastern Africa, and transmission risk is especially high for those living in close proximity to the Great Lakes of East Africa [23]. Some agricultural practices on adjacent land involving the use of certain pesticides can exacerbate schistosomiasis prevalence because of the detrimental effect these chemicals have on snail predators [24]. In addition, in some locations within Western Africa, there is evidence suggesting zoonotic transmission of *S. bovis* and *S. curassoni* from cattle, sheep, and goats to humans, with possible hybridization events involving *S. haematobium* [25]. This greatly complicates the transmission dynamics of *Schistosoma* in the presence of livestock.

In theory, the complete extirpations of snails from waterbodies would be sufficient to halt schistosomiasis transmission in humans and livestock. Yet, this objective is unattainable in practice. Treatments of transmission sites with Niclosamide, the molluscicide recommended by WHO (2017), can result in high mortality of snails [26,27]. Yet, its effect is only temporary (wettable formula not effective after 24 hours, [28]) as Niclosamide is subject to rapid photodegradation and it can be flushed away by water current [28]. Therefore, transmission sites can be rapidly recolonized by parasite-competent snails after treatment. In addition, the effect of this molluscicide is aspecific and causes massive mortality in the freshwater biodiversity, including fish, which are an essential source of protein and revenue for the local populations [29]. These limitations of chemical molluscicide use underscore the need for more sustainable and ecologically compatible methods for controlling snail vectors.

Biological control of parasite competent snails has been proposed as an alternative approach for the control of the parasite’s obligate intermediate host. In addition to bottom-up biological control through vegetation removal [9], use of snails’ natural enemies - such as catfish, river prawns or crayfish, all voracious predators of parasite-competent snails – have been proposed as agents for top-down biological control [10,30–33]. In spite of legitimate doubts about, and concern for, the effectiveness of top-down biological control of schistosomiasis [34], these studies have provided a compelling rationale and, in some cases, supportive evidence, for the use of snail’s natural enemies in schistosomiasis control. Moreover, the integration of aquaculture of commercially valuable species in schistosomiasis control [8] can pave the way to the widespread implementation of win-win planetary health solutions that support human health, improve nutrition and fight poverty [9].

## Study objectives

We investigated the effect of using African catfish, as a form of biocontrol, on snail vector abundance and *S. mansoni* infection intensity in SAC in lakeside communities. We used African catfish because they are endemic to Lake Victoria, they include freshwater snails in their diet [10], and are known to have declined with increased fishing and Nile perch introduction [35,36]. Using a Before-After-Control-Intervention (BACI) field study design, we tested whether the restoration of African catfish in December 2022, achieved through targeted stocking, was able to reduce the abundance of snail vectors and reinfection rates in SAC 12 months after intervention.

## Methods

**Fig 1:**
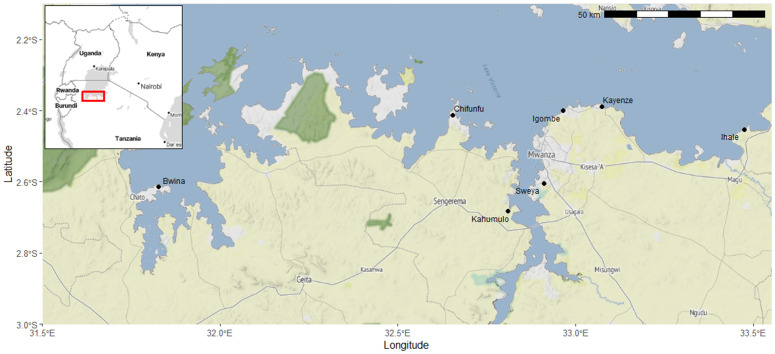
Map showing study region (southern shores of Lake Victoria, Tanzania). The seven study areas (control areas = Bwina, Igombe, Ihale, and Kayenze; intervention areas = Chifunfu, Kahumulo, and Sweya) are shown by black circles. Snail sampling sites (at known *S. mansoni* transmission sites associated with lakeside vegetation), fish intervention sites, and local primary schools were located within these areas. An inset map shows the broader Central African region with a red box indicating the location of the study area around Lake Victoria. Base map – Stamen Terrain tiles (Stamen Design via Stadia Maps, https://maps.stamen.com), used under CC BY 4.0 (https://creativecommons.org/licenses/by/4.0/). Boundaries – Natural Earth data (public domain, https://naturalearthdata.com). Overlays – Contains information from OpenStreetMap contributors and the OpenStreetMap Foundation (https://www.openstreetmap.org), available under the Open Database License (ODbL; https://www.openstreetmap.org/copyright).

This study was carried out in the Mwanza region of Tanzania, on the southern shores of Lake Victoria. *S. mansoni* is the predominant schistosome species in this region and the neighbouring lake zone, and transmission occurs in the lake or in habitats connected to the lake (Fig 1). Eleven snail surveys were conducted between December 2019 and November 2023 at 50 snail sampling sites across seven areas associated with high levels of *S. mansoni* transmission (Fig 1). Due to logistical constraints, not all sites were sampled during every survey, resulting in some variation in the number of surveys per site. Areas were selected based on local knowledge of human water contact activities (e.g., fishing, water collection, bathing) and the presence of high transmission risk.

**Fig 2:**
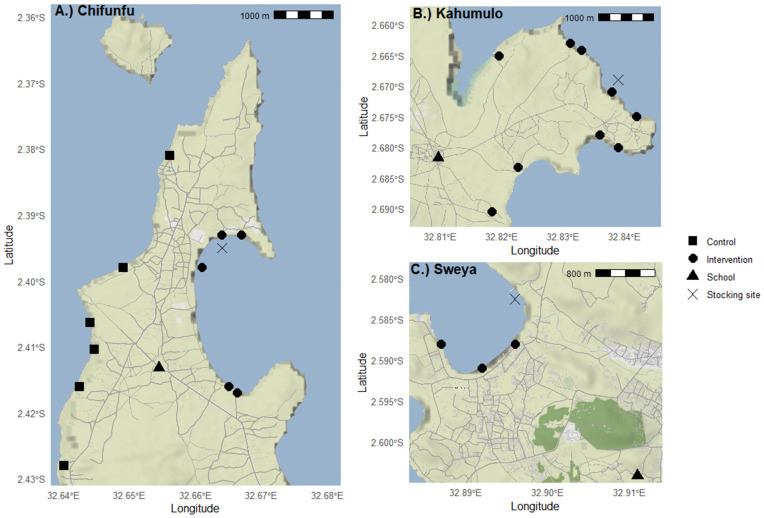
African catfish intervention sites, snail sampling sites and local primary schools in Chifunfu (A), Kahumulo (B) and Sweya (C). All snail sampling sites were designated as intervention sites in Kahumulo and Sweya. Due to the topography of Chifunfu and the location of the fish intervention site, only snail sampling sites on the eastern side of the peninsula were designated as intervention sites in Chifunfu. Fish intervention sites are shown by a black cross. Locations of primary schools, where human testing for *S. mansoni* was conducted, are shown by black triangles. Base map – Stamen Terrain tiles (Stamen Design via Stadia Maps, https://maps.stamen.com), used under CC BY 4.0 (https://creativecommons.org/licenses/by/4.0/). Boundaries – Natural Earth data (public domain, https://naturalearthdata.com). Overlays – Contains information from OpenStreetMap contributors and the OpenStreetMap Foundation (https://www.openstreetmap.org), available under the Open Database License (ODbL; https://www.openstreetmap.org/copyright).

African catfish were stocked at fish intervention sites in December 2022 located within three out of the seven areas (Chifunfu, Kahumulo, and Sweya; see Fig 2). These three areas were designated as ‘intervention’ areas and the other four as ‘control’ areas. Assignment of areas to treatment groups (intervention or control) was based on logistical feasibility for stocking African catfish (i.e., the release of catfish at specific intervention sites), habitat suitability, and local community acceptance, rather than randomization.

Human parasitology surveys were carried out at primary schools located within six out of the seven areas (one school per area; see Fig 2) in October 2022 (before intervention) and November 2023 (after intervention); however, due to logistical constraints, only five schools included both before and after surveys.

Snail surveys were conducted before the stocking intervention in November and December 2022, and post-stocking (i.e., after the stocking of catfish at intervention sites) in April and June 2023. Human parasitology surveys were carried out in October 2022 (pre-stocking) and in November 2023 (post-stocking).

Snail sampling sites and primary schools located within each area were designated as either ‘intervention’ or ‘control’ sites/schools based on the area designation (Fig 2). However, due to the topography of Chifunfu and the location of the fish intervention site (Fig 2), only snail sampling sites on the eastern side of the peninsula were designated as ‘intervention’ sites.

In total, there were 17 snail intervention sites (5 at Chifunfu, 3 in Sweya, and 9 in Kahumulo; see Fig 2) and 33 snail control sites (6 in Chifunfu, 8 in Igombe, 6 in Ihale, 10 in Bwina, and 3 in Kayenze).

To test the effect of stocking catfish at the fish intervention sites on snail vector abundance and human infection (prevalence and intensity), we applied a Before-After-Control-Intervention (BACI) design [37,38]. This widely used experimental framework in ecological studies compares changes over time between intervention and control groups to distinguish intervention effects from natural temporal variability. By monitoring snail sampling sites and schools in both control and intervention areas throughout the study period, the BACI design allowed us to compare conditions before and after the intervention, while accounting for natural environmental fluctuations.

### Snail surveys

Snail survey sites were chosen based on local information on human water contact activities, including fishing, water collection, and bathing. Snail surveys were conducted between December 2019 and November 2023, although surveys at individual sites were not strictly synchronized within each survey period due to logistical constraints.

Snails were surveyed in a quantitative manner by a team of people (team size varied between 1 and 5, but 70% of samples were collected by teams of 4) who scooped snails using metal sieve scoops (25 cm diameter, 0.5 mm mesh) on 140 cm poles for 15 minutes, which were then collected by hand using forceps and stored in a collection pot.

Whilst the sampling time (15 minutes at each site) remained constant throughout the study, the number of snail collectors varied, resulting in varying effort at each site (total effort = number of collectors x 15-minute search duration) and this is expected to impact the count data. Field teams also reported that there was not a constant and proportional relationship between observed counts and sampling effort. Sample positions were recorded using GPS.

All the snails were confirmed to be snail vectors (of the genus *Biomphalaria*) in the laboratory at the National Institute of Medical Research (NIMR), Mwanza Centre through shell morphology and then counted [39,40]. Snail sampling was carried out just before stocking catfish at the fish intervention sites in November and December 2022, and approximately 4–6 months after stocking, in April and June 2023.

To facilitate comparison between spatially mapped raw snail vector abundance, counts were standardised to an effort equal to four snail collectors (study mode value). This was achieved by first calculating the mean number of snails observed per person and then multiplying by 4. Site values were then averaged across periods (before and after stocking) and binned into categories based on the minimum and maximum counts and distribution quartiles.

### Human parasitology surveys

Human parasitology screening of SAC (aged 5–17 years) was carried out at six primary schools (excluding Kayenze) in October 2022 (pre-stocking) and repeated at five out of the six schools (excluding Igombe) in November 2023 (post-stocking). Infection prevalence (fraction of infected SAC in a school) and intensity (egg count) were tested by examining stool samples using the Kato-Katz method.

In 2022, stool samples were collected from each individual in two consecutive days and two Kato-Katz slides (41.7 mg of stool per slide) (WHO, 1994) were prepared from each stool sample (2 slides x 2 stools), and eggs counted for each slide. Repeat-counts enabled infection and egg detection probability to be estimated, as eggs are not always detected in a single stool sample of infected individuals. In 2023, four Kato-Katz slides were prepared on the same day from a single stool sample. The samples were transported to NIMR (Mwanza, Tanzania) laboratory for examination, where parasite eggs were counted by three experienced examiners (denoted as a, b, and c).

In total, 1,156 stools were tested by the three examiners (see Table A in S1 Text for more information). In 2022 (when stools were collected on consecutive days), stools were not collected for all SAC on the second day and hence fewer tests were carried out on day two in 2022. Egg counts were recorded for each slide and also summed by stool.

No antiparasitic treatments, such as praziquantel (PZQ), were administered to school children prior to stool collection to avoid biasing infection measurements. We did not collect systematic information on the use of traditional medicinal plants or foods with potential antiparasitic properties, and acknowledge this as a potential unmeasured factor. SAC found to be infected with *S. mansoni* were subsequently treated with a single 40 mg/kg oral dose of PZQ via the Tanzania National Neglected Tropical Diseases Control Programme, which is expected to reduce disease prevalence and/or egg burden.

### Stocking catfish at fish intervention sites

African catfish were raised at the Aquaculture Research and Development Centre in Kajjansi, near Entebbe, Uganda. In total, 50,000 fingerlings (c. 12 cm; 4 months old) were grown. These animals were anaesthetised (AquaSed, Vetark (2-phenoxyethanol)) and tagged using sub-dermal visible implant elastomer (Northwest Marine Technology, Inc.). This method was selected as it does not involve complicated application protocols or tag readers for identification upon re-capture; the tag can be seen directly. The tagging technique was also readily teachable to staff at Kajjansi, enabling tagging to be achieved rapidly despite the small size of the investigative team.

The tagged animals were returned to anaesthetic-free water to recover, and no acute mortality was observed. The animals were further rested until normal feeding and behaviour was resumed and no excess daily mortalities were observed.

The catfish were then transported by lorry and divided into equal portions for stocking at the three fish intervention sites in December 2022 (see Fig 2). Transport was achieved in 1,000 litre IBC pallet tanks. Oxygen was supplied via battery-powered air pumps and high-pressure cylinders. Two water changes of c. 50% were performed on route using borehole water. Upon stocking, mortality was below 1%.

Before restocking the local community was informed on the importance of protecting the catfish for up to three months post-stocking, before fishing them for food.

### Statistical methods

To assess the effectiveness of catfish stocking as a biocontrol intervention, we modelled three different response variables: (i) snail vector abundance, (ii) disease prevalence, and (iii) infection intensity (egg counts) in SAC. Each outcome required a tailored statistical approach, reflecting differences in data structure and ecological interpretation. All models included a BACI structure, with key predictors including treatment group (control vs intervention), time period (pre- or post-stocking), and their interaction.

The following sections outline the modelling framework applied to each outcome in detail.

### Snail count models

A generalised linear mixed model (GLMM) was used to test the effect of stocking catfish at the fish intervention sites on site-level snail counts. ‘Site-type’ (i.e., control vs intervention sites, where catfish were restored) and ‘Period’ (before or after stocking) were set as fixed factors, and the interaction term ‘Site-type:Period’ was used to test whether the before-after difference in snail vector abundance could be attributed to catfish intervention (see Table 1).

**Table 1:** Predictors used in the modelling analysis. The ‘Snail count’ model refers to the Generalised Linear Mixed Model (GLMM) used to model snail counts, ‘Prevalence’ refers to the disease prevalence models, ‘Egg count’ refers to the GLMM used to model infection intensity (egg count) in school-aged children (SAC) stools. Some models included a zero-inflation (zi) component to account for excess zeros. A single tick (✓) indicates that the predictor was included in the main (conditional) component of the model; a double tick (✓✓) indicates inclusion in both the conditional and zero-inflation components.

Predictor	Model usage	Variable Type	Description	Snail count	Prevalence	Egg count
Site-type	Fixed	Dichotomous variable	Values: ‘Control’, ‘Intervention’ (site).	✓	✓	✓
Period	Fixed	Dichotomous variable	Values: ‘Before’, ‘After’ (stocking).	✓	✓	✓✓
Site-type:Period	Fixed	Interaction	Interaction between Site-type and stocking period.	✓	✓	✓
Area	Fixed/ Random	Categorical variable	Area unique name (Bwina, Chifunfu, Igombe, Ihale, Kahumulo, Kayenze, and Sweya).		✓	✓✓
Site	Fixed	Categorical variable	Snail site unique name.	✓		
Examiner	Fixed	Categorical variable	Stool examiner (‘a’,’b’ or ‘c’).		✓	✓✓
Scoop-effort	Fixed	Continuous	Total person minutes (time searching for snails x number of people searching).	✓✓		
Slides	Offset	Discrete	Number of slides examined.			✓
Test	Fixed	Categorical variable	Test label used when data stacked by test (1 = slides 1 and 2 collected in 2022; 2 = slides 1 and 2 collected in 2023; 3 = slides 3 and 4 collected in 2023).		✓	

To account for between site variability in snail counts, a site-level random effect was added (i.e., intercepts vary by site). Typically, the inclusion of a random effect would not impact the size of the estimated stocking effect (but would likely impact the interpretation of the other fixed effects), but in this study, some sites were surveyed more than others, introducing potential site-level biases. The random effect term in the model helped control for this.

We accounted for varying effort by including an explanatory variable ‘Scoop-effort’ as a fixed effect in the model. Scoop-effort was included as a fixed effect, rather than as an offset, because field observations indicated that increases in sampling effort did not result in strictly proportional increases in snail counts.

The snail count data were found to be zero-inflated, i.e., including more zeros than expected according to a Poisson or negative binomial distribution. The probability of generating excess zeros (beyond that expected to be generated by the sampling process) was modelled as a separate ‘zero-inflated’ (zi) component in the GLMM, comprising a binomial GLM with a logit link function. For the purposes of this snail count model, we assumed that the excess zeros were generated from variability in site occupancy; note that the true source of the excess zeros is not important here, since we are focused on the effect on positive snail counts. Since variability in effort will impact site occupancy, ‘Scoop-effort’ was added as a fixed effect into the zero-inflated model. Whilst imperfect detection will likely bias snail counts low, it will not impact the estimated effect size (or significance) of fish intervention, as detection-related zeros are handled by the model’s error structure.

Site-level random effects were included in the generalized linear mixed model (GLMM) to account for consistent differences among sites, but temporal autocorrelation or correlations among measurements within or between survey periods were not explicitly modelled. The primary analytical goal was to test the intervention effect using a BACI structure, comparing overall pre- and post-stocking periods across intervention and control sites. Snail count modelling was carried out using the statistical software programming language R (version 4.4.1) [41] using the ‘glmmTMB’ package (version 1.1.8) [42,43].

### Human parasitology models

#### Disease prevalence models.

Disease prevalence can be modelled using occupancy analysis, which accounts for variability in detection probability. In this framework, the individual – here, the stool sample - is treated as the ‘site’ and the parasite as the site occupant. Therefore, the proportion of infected SAC can be viewed as site occupancy, or disease prevalence.

In this study, disease prevalence and disease detection probability were studied at the individual (i.e., between stool) level. We fitted the single-season occupancy model of MacKenzie (2002) [44] to test disease detection probability in SAC, using two Kato-Katz slides per stool. The four slides examined for the 2023 data were split into two sets of two slides each, and each set was treated as an independent test (i.e., entered as a separate row in the dataset) to match the structure of the 2022 data.

When stacking data in this way to include all observations in the model, the assumptions of independence between tests is often violated. However, this typically has a limited effect on results, particularly when a stacking factor variable (‘Test’, see Table 1) is included as a fixed effect (see [45]). The ‘Test’ variable was included explicitly as a nuisance factor to control for procedural differences between sampling protocols (i.e., two stools collected in 2022 versus one stool in 2023), and was not biologically interpreted.

We used a candidate model approach to evaluate predictors of disease prevalence, with model selection based on AIC. Candidate models incorporated biologically relevant predictors such as Site-type, Period, Site-type:Period interaction, Area, Examiner, and Test. Outputs were interpreted cautiously, recognizing that model selection and inference were performed on the same dataset.

Modelling was carried out using the ‘occu’ function in the ‘unmarked’ R package (version 1.4.1) [46,47]. MacKenzie and Bailey (2004) [48] goodness-of-fit tests (based on Pearson chi-square using 1000 bootstrap samples) and AIC were used to assess model fit to the data.

### Egg count models

Preliminary analysis showed that egg counts differed significantly between replicate Kato-Katz slides (Wilcoxon signed-rank test). Therefore, the egg count data were analysed at the stool-level (i.e., data were stacked by stool and egg counts summed across slides) using a GLMM; a N-mixture model as described by Royle (2004) that accounted for detection probability was also tested in preliminary analysis, but model fit to the data was poor.

The final GLMM included a zero-inflation component to account for variation in disease prevalence, and an offset was applied to control for effort (number of slides, between one and four). The zero-inflated component modelled the probability of structural zeros (true uninfected individuals or undetected infections), while the conditional component modelled infection intensity (egg counts) using a negative binomial distribution. This approach allowed us to retain all available data while appropriately modelling both infection probability and infection intensity.

A BACI model structure was used to examine the effect that stocking catfish at the fish intervention sites had on egg counts, where ‘Site-type’ and ‘Period’ were modelled as fixed effects and the interaction term (‘Site-type:period’) was used to determine the significance and size of the stocking effect (see Table 1 for predictors).

Modelling was carried out using the ‘glmmTMB’ function in the ‘glmmTMB’ R package (version 1.1.8) [42,43]. Model fit to the data was assessed using the ‘DHARMa’ R package (version 0.4.6) [49].

During preliminary modelling analyses, adding Examiner as a fixed effect to the model improved fit but produced an implausibly large effect size, likely reflecting confounding between Examiner and Site-type:Period (as Examiner ‘c’ primarily assessed post-stocking intervention samples). Adding Area as either a random effect or fixed effect resulted in poor model fit or rank deficiency, due to the small number of areas and overlap with intervention status. Consequently, Examiner and Area were not included in the final egg count models.

### Ethical considerations

Ethical clearance was obtained from the Institutional Review Board (IRB) of the University of St Andrews and the National Institute of Medical Research (NIMR), Mwanza Centre (Permit number NIMR/HQ/R.8a/Vol. IX/4155) and sample collections and examination were performed in accordance with study protocol. Permissions to conduct the study were also obtained from the Regional and District Medical Offices, as well as from Headteachers of participating schools. School teachers, parents/guardians, and SAC were informed about the purposes, procedures, and usefulness of the study. Only SAC who were willing to participate in the study and whose parents or legal guardians signed the informed consent forms were allowed to participate in the study.

## Results

### Snail counts

The mean proportion of sites occupied by snail vectors (not accounting for variation in sampling effort) over the study period ranged from 0 to 1 and mean counts (for occupied sites) ranged from 4 to 230.6 snails (Table 2). Raw snail counts reduced at intervention sites after stocking catfish in both Chifunfu and Kahumulo and remained stable in Sweya (Fig A in S1 Text). In Chifunfu, raw mean snail counts for the 3 occupied intervention sites all reduced after stocking (Fig B in S1 Text). In Kahumulo, mean counts at 8 out of the 9 intervention sites reduced after stocking (Fig C in S1 Text) and in Sweya, mean counts at 2 out of the 3 intervention sites reduced after stocking (Fig D in S1 Text). During the study, 7 out of the 50 sites were never occupied by snail vectors (3 in Chifunfu, 2 in Bwina and 2 in Ihale). Snail vectors were not found in 11 sites before stocking and 16 sites after stocking (note that not all sites were sampled during every snail survey and hence raw snail vector occupancy results will likely be biased by sampling effort).

**Table 2:** Summary of snail survey effort and count data. Occupancy and counts are given as area averages across site means, with associated site mean ranges given in brackets. Note that both control and intervention sites were sampled in Chifunfu.

Area	Site type	Surveys	Sites	Total sites sampled	Mean proportion of sites occupied	Mean site count (occupied sites)
Bwina	Control	11	10	82	0.61 (0 to 1)	53.11 (4 to 91.4)
Chifunfu	Control	11	6	45	0.74 (0 to 1)	107.58 (69.75 to 160)
Chifunfu	Intervention	11	5	27	0.53 (0 to 1)	58.8 (50.33 to 74.5)
Igombe	Control	10	8	53	0.73 (0.5 to 1)	96.72 (25 to 230.6)
Ihale	Control	11	6	33	0.60 (0 to 1)	90.11 (47.3 to 138.33)
Kahumulo	Intervention	11	9	83	1.00 (1 to 1)	85.27 (59.11 to 121.4)
Kayenze	Control	10	3	27	0.61 (0.38 to 1)	46.68 (13 to 82.8)
Sweya	Intervention	7	3	13	0.78 (0.33 to 1)	84.19 (20 to 171)

### Snail count model

**Fig 3:**
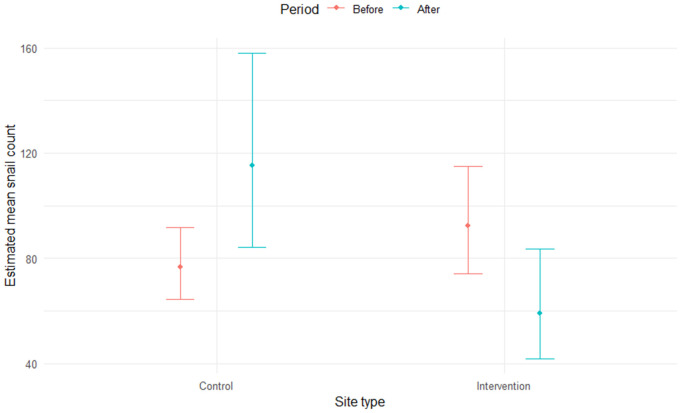
Estimated mean snail counts for occupied sites (estimations made using conditional model, not including zero-inflated component). Error bars indicate 95% confidence intervals for mean counts.

The snail count model was fit to 363 snail counts collected during 11 surveys (between 2019 and 2023) across 50 different sites. Based on the fitted model parameter estimates and associated standard errors and *p* values (Table B in S1 Text), stocking catfish at the fish intervention sites had a significant negative impact on snail count (see ‘Intervention:After’ interaction term in Table B in S1 Text, and also Fig 3). Although no difference in estimated mean snail count between control and intervention sites was found (see non-significant ‘Impact’ term in Table B in S1 Text), a significant positive effect on mean snail count was found for the ‘After’ period. As expected, the ‘Scoop-effort’ fixed effect variable was found to significantly increase snail counts and also significantly decrease the probability of generating excess zeros in the zi model (i.e., that an increase in effort increased the probability of detecting snail vectors when present).

The coefficient value (on the scale of the log link function) for the interaction term in the count model was -0.85, which equates to an estimated 0.43 (e^-0.85^) factor change (or 57% decrease, 95% CI: 29.4%, 74.3%) in mean site-level snail count at occupied sites when catfish were stocked at the fish intervention sites.

### Human parasitology surveys

Observed disease prevalence (not considering sampling biases) in SAC ranged between 0.07 and 0.81 in 2022 (*n* = 6) and between 0.03 and 0.44 in 2023 (*n* = 5). Mean disease prevalence was on average lower in 2023 than in 2022 (Table 3 and Fig E in S1 Text). This reduction in disease prevalence is partially due to the fact that two stools were collected for each individual in 2022 and only one in 2023 (Table 3). The study-wide decrease in prevalence was also observed when analysing individual stools (i.e., taking a mean across the two stools collected in 2022, instead of combining the test results), except at Kahumulo, where the mean prevalence in 2022 and 2023 were the same. The most substantial change in observed disease prevalence was a decrease of 52.7% at the Chifunfu primary school (Table 3). Prevalence was also compared between slides using a paired McNemar test. No significant differences between slides (or stool samples) were found (i.e., all *p* values were found to be > 0.05).

**Table 3:** Summary of results for human parasitology testing at primary schools on school-aged children (SAC) across the study region. In 2022, two stools (S1 and S2) were collected per SAC where possible. Results are presented for each individual stool (S1 or S2 separately) and for combined results across stools (S1 + S2). Percentage change between 2022 and 2023 is given for both prevalence and mean eggs using mean stool-based values (not combined SAC-level values). Not all SAC provided a second stool in 2022. SAC at Igombe primary school were only tested in 2022 and no tests were conducted in Kayenze.

		Prevalence				Infection intensity: Mean number of eggs (per gram of stool)			
Area	School type	2022 (S1, S2)	2022 S1 + S2	2023 S1	Change (%)	2022 (S1, S2)	2022 SAC	2023 S1	Change (%)
Bwina	Control	0.43 (0.45, 0.41)	0.57	0.38	-12	79 (86, 73)	60	46	-42
Chifunfu	Intervention	0.74 (0.74, 0.74)	0.81	0.35	-53	133 (138, 127)	121	71	-46
Igombe	Control	0.49 (0.51, 0.47)	0.7			80 (97, 63)	56		
Ihale	Control	0.6 (0.62, 0.57)	0.65	0.44	-27	276 (280, 271)	247	210	-24
Kahumulo	Intervention	0.19 (0.15, 0.22)	0.28	0.19	0	53 (50, 5550,55)	34	35	-33
Sweya	Intervention	0.05 (0.04, 0.05)	0.07	0.03	-40	41 (45, 3636,45)	23	39	-44

Infection intensity (mean eggs per gram of stool) in SAC decreased in all schools after stocking except for in Sweya and Kahumulo (Fig E in S1 Text) and ranged between 23 eggs/g (Sweya, 2022) and 247 eggs/g (Ihale, 2022). However, since infection is not always detected in stools, combining counts across multiple stools (as in 2022) may reduce variability and lower mean counts compared to single-stool sampling (as in 2023). On the other hand, differences in sampling protocol could lead to either higher or lower observed mean counts, depending on day-to-day variation in egg output. When comparing means across stool tests, mean egg counts reduced in all schools (Table 3). As with prevalence, the infection intensity reduced most substantially at the Chifunfu primary school.

### Disease prevalence models

**Fig 4:**
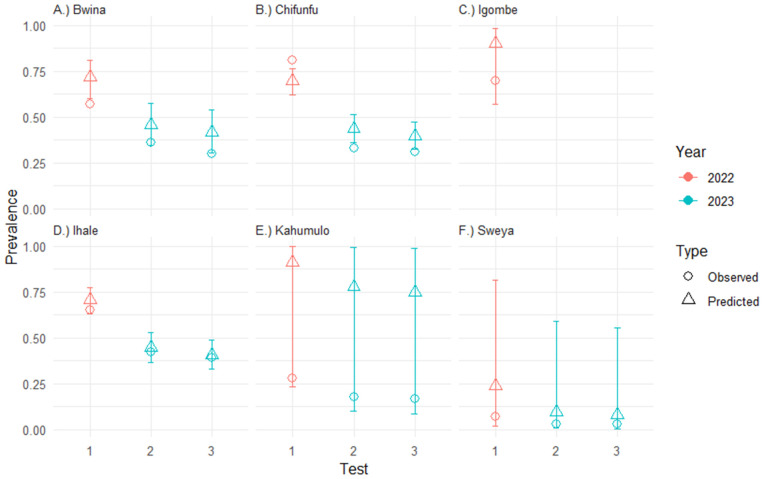
Estimated and observed disease prevalence in school-aged children by area, test and sampling year. Note that the four slides collected in 2023 (Tests 2 and 3) for a single stool were split into two tests to enable comparison with 2022 (Test 1) data. Error bars indicate 95% confidence intervals for estimated prevalence values.

In total, 1712 test results were included in the prevalence model (600 in 2022 and 1,112 in 2023). The best model (with the lowest AIC) from the candidate model set included ‘Area’ as a predictor for the detection model component and ‘Area’ and ‘Test’ as predictors for the prevalence model component (Fig 4). MacKenzie and Bailey (2004) goodness-of-fit test indicated that the model fit to the data was good (*Χ^2^* = 1.5, *p* = 0.26, *ĉ* = 1.24). The effect of stocking on disease prevalence for a BACI model (Site-type*Period, without control for ‘Test’) was found to be significant and negative (*β* = -0.70, SE = 0.34, *p* = 0.039) but model fit to data was poor, indicated by the low value as determined from the MacKenzie and Bailey (2004) goodness-of-fit test (*Χ^2^* = 0.54, *p* = 0.65, *ĉ* = 0.4).

Based on the results of the prevalence modelling analysis, we found a substantial decrease in prevalence between 2022 and 2023. However, there was considerable uncertainty in estimations at the Kahumulo and Sweya intervention schools (Fig 4E and 4F).

### Infection intensity model

**Fig 5:**
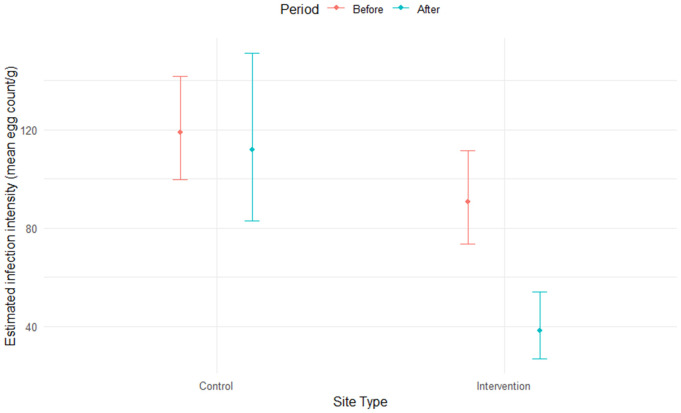
Estimated infection intensity (mean egg count per gram) for infected stools. Estimations were made using conditional model, not including zero-inflated component. The model indicates that stocking had a significant negative effect (*p* = 0.0018) on infection intensity. Note that the model included an offset term for effort (‘Slides’) and prevalence was controlled by a zero-inflation component (‘Area + Period’).

Model fit for the BACI infection intensity model was assessed using the ‘DHARMa’ R package [50,49] and found to be adequate, with no evidence of distributional violations (Kolmogorov–Smirnov test, *p* = 0.19), overdispersion (*p* = 0.11) or influential outliers (*p* = 0.40) [44]. Stocking catfish (assessed via ‘Site-type:Period’ interaction term) was found to have a significant (*p* = 0.0018) negative effect on infection intensity (Fig 5 and Table C in S1 Text), reducing mean parasite egg counts by 55% (95% CI: 26%, 73%) for infected SAC.

## Discussion

Control of schistosomiasis calls for a multi-modal form of intervention. While seasonal chemotherapy campaigns can reduce infection rates effectively in some settings, in areas where communities are in frequent contact with year-round standing water, such as along rivers and lakes, rapid reinfection due to ongoing water exposure can limit the long-term success of chemotherapy alone. In this study, we found that restoring catfish at fish intervention sites significantly reduced mean site-level counts of snail vectors at occupied sites by 57% (95% CI: 29.4%, 74.3%) and had a significant negative effect on SAC infection intensity, reducing mean counts by 55% (95% CI: 26%, 73%). The largest decrease in disease prevalence (-52.7%), infection intensity (-46.14%) and snail count (-80%) was found in the Chifunfu area, where catfish were stocked at a fish intervention site. We also found that both disease prevalence and infection intensity (egg count) in SAC reduced in all areas. These results suggest that stocking catfish may provide an effective means of biocontrol for snail vectors, but further investigations are needed. Breaking the infection cycle will, however, require the removal of both the source of infection for host snails and for people. It therefore appears that there may be effective methods to do both, implementing a strategy of preventative chemotherapy for local human populations and biological control for host snails. A key to biological control, however, will be to create incentives for local fishers to sustain snail predators, possibly by developing them as a culture to supply food either for local use or for export.

The restoration of a native predator (African catfish) at inland sites around Lake Victoria may not only benefit human health but may also provide fishing opportunities (as long as the fishing is appropriately managed). This would serve to both increase economic resilience in the region, through export, and bolster the health and wellbeing of lakeside communities, since diets associated with high fish content (and added nutrition) have been linked to good health [51]. If effective aquaculture practices can be developed alongside growth in the fishing industry, culture of African catfish could potentially provide a long-term solution to high infection intensity presently observed in lake side communities. This is particularly important considering the WHO latest guidelines, which underline the importance of tackling both prevalence and intensity [22]. Of course, history is replete with examples of human modification of ecosystems that have had detrimental effects on both wildlife and humans, and Lake Victoria is no exception, with the disastrous consequences for fish species diversity after the introduction of Nile Perch [52]. In fact, human activities around the lake such as irrigation and removal of snail predators has already had an effect on the ecosystem. These impacts may be somewhat perturbed by increasing temperatures as the climate changes and snail ranges reduce. In any case, using a native species for such experiments limits risks to some extent and clearly offers benefits to local communities. Further, if such a biocontrol method was to be implemented, the costs are expected to be far less than the alternative, which are intensive praziquantel campaigns that could eventually be challenged by the emergence of drug resistance [53]. Coupling regular, seasonal treatment with biocontrol may help mitigate to some extent the population resistance to the drug.

African catfish may serve as effective biocontrol agents by reducing exposure to *S. mansoni* infection through decreasing the abundance of parasite-competent *Biomphalaria* snail vectors in nearshore lake habitats. However, *S. haematobium*, which is transmitted by *Bulinus spp*., is also highly prevalent in inland ponds and springs throughout this region [54,55]. While catfish may be unsuitable for use in these small, temporary waterbodies, other predators, such as *Macrobrachium* prawns could be considered. While these prawns are not native to the region, they have been highly developed as an aquaculture species and can be reared in hatcheries and disseminated to putative transmission sites [56]. Importantly, they require estuarine water to reproduce, and therefore pose extremely low risk of invasion in the region surrounding Lake Victoria, which is several thousand kilometers from suitable reproductive habitat. Nevertheless, any introduction of non-native species should proceed cautiously, with thorough ecological risk assessment, even when biological constraints suggest minimal invasion potential. Alternatively, native predators could be evaluated for these inland sites. Freshwater crabs, water scorpions, dragonflies, damselflies, and other taxa are present in these sites (personal observations, Angelo, Civitello, and Kinung’hi), and preliminary laboratory observations suggest that these taxa can prey upon *Bulinus nasutus* snails.

There were several limitations associated with the study, mainly related to data collection, site coverage and protocol consistency.

Variability between areas and examiner effects could not be controlled for in the infection intensity model due to data limitations, so unfortunately at this time we cannot infer the effect of catfish stocking (or its relationship with the effect of stocking on snail count) outside of the study area. Interestingly, the estimated reductions in snail occupancy and infection intensity were of similar magnitude (~55–57%), although given the variability and study limitations, this may be coincidental rather than indicative of a direct mechanistic relationship.

Although no large-scale mass drug administration (MDA) programs were conducted during the study period in the study areas, other localized interventions such as health education or community-led WASH (Water, Sanitation and Hygiene) initiatives may have contributed to the observed reductions in infection prevalence and intensity. WASH practices were not systematically recorded during the study. Differences in WASH access and behaviour between communities could influence schistosomiasis transmission dynamics and may have contributed to observed infection trends independently of the intervention. Therefore, we cannot attribute the reductions exclusively to the fish stocking intervention.

The study included snail sampling in seven areas, but due to logistical constraints, human parasitology only occurred within five of the seven areas, before and after stocking catfish at the fish intervention sites. Since this was not a cohort study, only disease prevalence and intensity (egg count) could be monitored (not incidence). Prepatent infection prevalence in snails was also not monitored in this study. This could be included in future studies to provide a better idea of number of snails that are being infected, rather than a snapshot of actively shedding snails.

Sampling protocols for human testing changed between 2022 and 2023, from testing two slides per stool for two stools (on consecutive days) in 2022, to testing four slides from a single stool in 2023. This change in protocol limited what types of analysis and modelling could be carried out and also likely contributed to poor model fit to the data. Currently our infection intensity model should not be used to make population inference. Whilst the results of our inflection intensity model are promising, showing a stocking effect reducing infection intensity, these results only hold within the range of data within the model and cannot be used to infer to other areas. Ideally, we would like to collect data from more areas, enabling us to model area as a random effect. We would also like to account for examiner effect, but to do so we need to send examiners to multiple site types during multiple periods (sampling each combination of the BACI design).

Although our site-level random effects accounted for consistent differences among sites, our analysis did not explicitly model temporal autocorrelation or potential correlations between samples taken closely in time. Additionally, snail sampling dates were not fully synchronized across sites due to logistical limitations. An interrupted time-series or another temporally explicit modelling approach might have provided additional insights into finer-scale temporal dynamics, but would not address our primary goal of evaluating the intervention’s overall effect across multiple sites.

Intervention areas were geographically clustered within ~50 km of each other, increasing the possibility that localized environmental events (e.g., flooding, pathogen outbreaks) could have influenced snail populations independent of the stocking intervention. Furthermore, the ‘before’ and ‘after’ snail surveys were conducted at different times of the year, corresponding to distinct climatic seasons. In the Mwanza region, rainfall typically peaks between March and May during the ‘long rains’ season, potentially altering habitat suitability and snail abundance. While the study’s BACI design and the use of contemporaneous control areas provide defences against regional environmental confounding, localized environmental variability between intervention and control areas cannot be fully excluded. Future studies should incorporate direct meteorological monitoring to better distinguish intervention effects from seasonal or climatic variation. Indeed, with sufficiently high samples (to maintain sufficient degrees of freedom), the effect of local environmental variability could be explicitly considered in future modelling.

Finally, given the number of fish released and the high suitability of surrounding habitat, it was expected that a substantial proportion of the fish remained close to the fish intervention sites. However, we acknowledge that habitat suitability gradients were not directly surveyed, and that catfish may have dispersed more widely than assumed, especially across transitional habitats. Although 10% of the stocked fish were tagged, none were retrieved, due to either apparent mortality or unreported catches by local fishers. This lack of direct fish monitoring represents a major limitation, making it difficult to conclusively attribute observed reductions in snail abundance and infection intensity to catfish presence alone. Future studies should incorporate systematic fish population surveys, ideally with incentives for fishers to report tag recoveries, to strengthen causal inference between predator restoration and schistosomiasis transmission dynamics.

## Conclusions

We found that restoring catfish in the Tanzanian sector of Lake Victoria likely reduced schistosomiasis infection intensity in SAC, by reducing the number of snail vectors at known transmission sites. Scaling up this approach to lake-wide schistosomiasis control will require systemic intervention tailored to circumstances. The next steps include developing a model of disease dynamics to better understand how catfish (and other predators) could be used in practice. During this study, we learnt that moving live fish hundreds of kilometres by road is very challenging and perhaps not a viable solution for future work. An alternate approach would be to grow fish local to stocking sites. For example, the Tanzanian Fisheries Research Institute aquaculture facility in Mwanza could be refurbished, to produce African catfish. While a focus on fish culture is reasonable, making sure this is seen as one part of a “One Health” system of intervention – involving bilateral discussions between researchers and community members about hygiene, regular chemotherapy, and potential habitat management is also important.

## Supporting information

S1 Code R script used for modelling analyses. This R script includes the complete code for fitting and evaluating the three main statistical models used in the study: Snail count model (zero-inflated negative binomial GLMM using glmmTMB). Human disease prevalence model (occupancy model using unmarked). Infection intensity model (zero-inflated negative binomial GLMM using glmmTMB).
(R)

S1 Data Snail count data. Contains snail count data collected from 50 sites across 11 surveys between 2019 and 2023. Key columns include: Site.name (unique site ID), Period (before or after intervention), Site.type (intervention or control), Snail.count (number of Biomphalaria snails found), and Scoop.effort (relative sampling effort).
(CSV)

S2 Data Human parasitology prevalence data. Contains binary prevalence data from school-aged children (SAC) for use in occupancy modelling. Key columns include: Site.type (intervention or control), Period (before or after stocking), Area (study area), and Test (detection/non-detection results for each test occasion).
(CSV)

S3 Data Human parasitology infection intensity data. Contains egg count data for school-aged children (SAC) for infection intensity modelling. Key columns include: Site.type (intervention or control), Period (before or after stocking), Area (study area), Egg.counts (number of eggs counted), and Slides (number of slides examined).
(CSV)

S1 Text A file that contains Figures A-E and Tables A-C, described below. **Fig A. Raw observed snail (*Biomphalaria spp.*) counts by area, period (before or after stocking) and site type (control or intervention site).** Samples size indicated below x-axis. For B.), sample size is the sum of the counts recorded at the control and intervention sites. Since the raw count values are biased by snail detection probability and uneven sampling effort over time and across sites, they cannot be used to reliably assess the impact of stocking catfish. **Fig B. Mean snail counts observed in the Chifunfu area by site type and period.** Due to the topography of the area, sites were split into intervention sites close to the fish intervention site (catfish stocking locations) on the eastern side of the peninsula and control sites on the western side. Numbers in (B) represent change in mean count between pre- and post-stocking periods. Snail count categories were defined as: not occupied = 0, low = 0–44, medium = 44–111, and high = 111–241 snails. Note that some sites were never occupied by snail vectors. Base map – Stamen Terrain tiles (Stamen Design via Stadia Maps, https://maps.stamen.com), used under CC BY 4.0 (https://creativecommons.org/licenses/by/4.0/). Boundaries – Natural Earth data (public domain, https://naturalearthdata.com). Overlays – Contains information from OpenStreetMap contributors and the OpenStreetMap Foundation (https://www.openstreetmap.org), available under the Open Database License (ODbL; https://www.openstreetmap.org/copyright). **Fig C. Mean snail counts observed in the Kahumulo area by site type and period.** All sites in this area were considered to be intervention sites due to their proximity to the fish intervention site (catfish stocking locations). Numbers in (B) represent change in mean count between pre- and post-stocking periods. Snail count categories were defined as: not occupied = 0, low = 0–44, medium = 44–111, and high = 111–241 snails. Base map – Stamen Terrain tiles (Stamen Design via Stadia Maps, https://maps.stamen.com), used under CC BY 4.0 (https://creativecommons.org/licenses/by/4.0/). Boundaries – Natural Earth data (public domain, https://naturalearthdata.com). Overlays – Contains information from OpenStreetMap contributors and the OpenStreetMap Foundation (https://www.openstreetmap.org), available under the Open Database License (ODbL; https://www.openstreetmap.org/copyright). **Fig D. Mean snail counts observed in the Sweya area by site type and period.** All sites in this area were considered to be intervention sites due to their proximity to the fish intervention site (catfish stocking locations). Numbers in (B) represent change in mean count between pre- and post-stocking periods. Snail count categories were defined as: not occupied = 0, low = 0–44, medium = 44–111, and high = 111–241 snails. Base map – Stamen Terrain tiles (Stamen Design via Stadia Maps, https://maps.stamen.com), used under CC BY 4.0 (https://creativecommons.org/licenses/by/4.0/). Boundaries – Natural Earth data (public domain, https://naturalearthdata.com). Overlays – Contains information from OpenStreetMap contributors and the OpenStreetMap Foundation (https://www.openstreetmap.org), available under the Open Database License (ODbL; https://www.openstreetmap.org/copyright). **Fig E. Mean number of eggs per gram of stool for infected school-aged children (SAC) tested before and after stocking catfish based on raw observations (not considering sampling biases).** Disease prevalence indicated below x-axis. The solid dots indicate outliers (1.5 times the interquartile range above the third quartile or below the first quartile). SAC at Igombe primary school were only tested in 2022 and no tests were conducted in Kayenze. **Table A. Sample size of human parasitology tests carried out by the three examiners (a, b and c) at primary schools on school-aged children (SAC) in October 2022 and November 2023**. In 2022, a stool sample was obtained on consecutive days (S1 and S2). In 2023, only a single stool was tested. Not all SAC provided a second stool in 2022. SAC at Igombe primary school were only tested in 2022 and no tests were conducted in Kayenze. **Table B. GLMM model results (fixed effects only).** *β* is the model parameter estimate, SE is the standard error and zi is the zero-inflation component of the model (i.e., a binomial GLM used to estimate probability of generating excess zeros). **Table C. GLMM egg model results (conditional model only).** *β* is the model parameter estimate, and SE is the standard error.
(DOCX)
