# Supplementary material for: Stocking African catfish in Lake Victoria provides effective biocontrol of snail vectors of Schistosoma mansoni
Source: PLoS Negl Trop Dis. 2025 Sep 3;19(9):e0013490. doi: 10.1371/journal.pntd.0013490 (PMC12419634; doi:10.1371/journal.pntd.0013490)
Supplement: S1 Text — Fig A. Raw observed snail (Biomphalaria spp.) counts by area, period (before or after stocking) and site type (control or intervention site). Samples size indicated below x-axis. For B.), sample size is the sum of the counts recorded at the control and intervention sites. Since the raw count values are biased by snail detection probability and uneven sampling effort over time and across sites, they cannot be used to reliably assess the impact of stocking catfish. Fig B. Mean snail counts observed in the Chifunfu area by site type and period. Due to the topography of the area, sites were split into intervention sites close to the fish intervention site (catfish stocking locations) on the eastern side of the peninsula and control sites on the western side. Numbers in (B) represent change in mean count between pre- and post-stocking periods. Snail count categories were defined as: not occupied = 0, low = 0–44, medium = 44–111, and high = 111–241 snails. Note that some sites were never occupied by snail vectors. Base map – Stamen Terrain tiles (Stamen Design via Stadia Maps, https://maps.stamen.com), used under CC BY 4.0 (https://creativecommons.org/licenses/by/4.0/). Boundaries – Natural Earth data (public domain, https://naturalearthdata.com). Overlays – Contains information from OpenStreetMap contributors and the OpenStreetMap Foundation (https://www.openstreetmap.org), available under the Open Database License (ODbL; https://www.openstreetmap.org/copyright). Fig C. Mean snail counts observed in the Kahumulo area by site type and period. All sites in this area were considered to be intervention sites due to their proximity to the fish intervention site (catfish stocking locations). Numbers in (B) represent change in mean count between pre- and post-stocking periods. Snail count categories were defined as: not occupied = 0, low = 0–44, medium = 44–111, and high = 111–241 snails. Base map – Stamen Terrain tiles (Stamen Design via Stadia Maps, https://maps.stamen.com), [file pntd.0013490.s005.docx]

# S1 Text


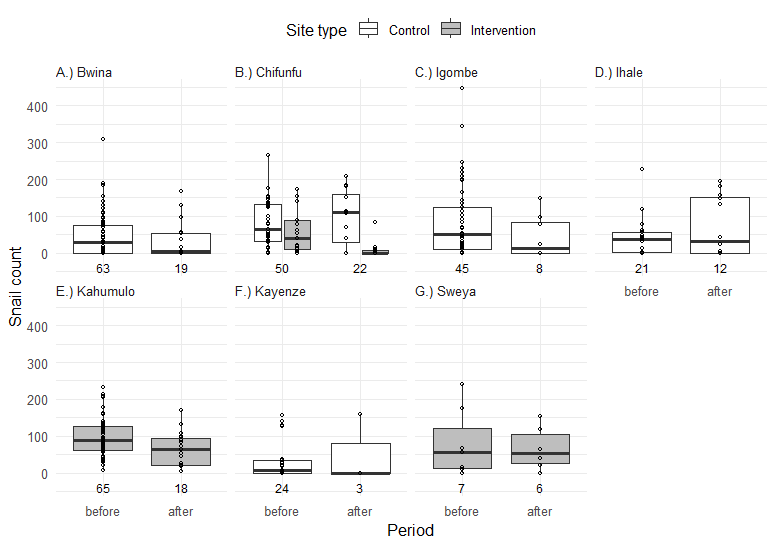


Fig A. Raw observed snail (*Biomphalaria spp.*) counts by area, period (before or after stocking) and site type (control or intervention site). Samples size indicated below x-axis. For B.), sample size is the sum of the counts recorded at the control and intervention sites. Since the raw count values are biased by snail detection probability and uneven sampling effort over time and across sites, they cannot be used to reliably assess the impact of stocking catfish.


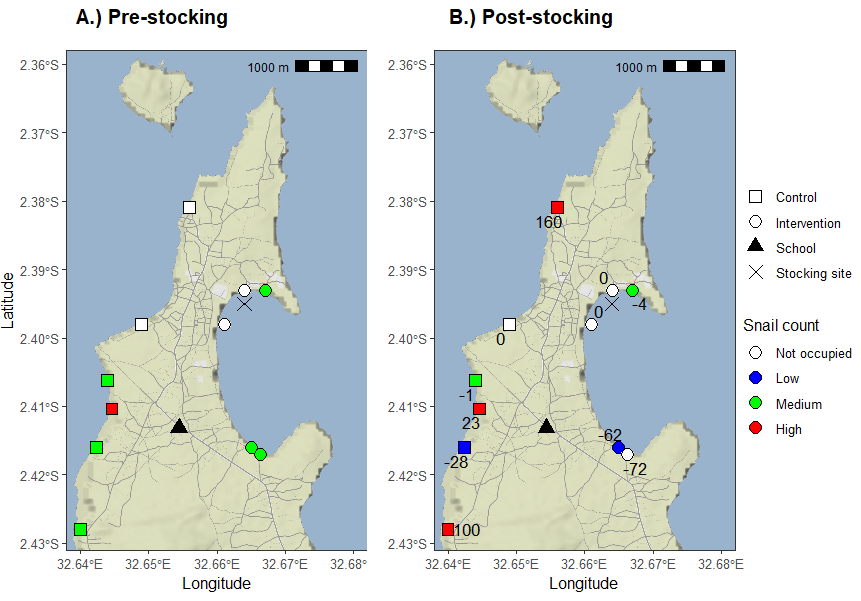


Fig B. Mean snail counts observed in the Chifunfu area by site type and period. Due to the topography of the area, sites were split into intervention sites close to the fish intervention site (catfish stocking locations) on the eastern side of the peninsula and control sites on the western side. Numbers in (B) represent change in mean count between pre- and post-stocking periods. Snail count categories were defined as: not occupied = 0, low = 0-44, medium = 44-111, and high = 111-241 snails. Note that some sites were never occupied by snails. Base map – Stamen Terrain tiles (Stamen Design via Stadia Maps, https://maps.stamen.com), used under CC BY 4.0 (https://creativecommons.org/licenses/by/4.0/). Boundaries – Natural Earth data (public domain, https://naturalearthdata.com). Overlays – Contains information from OpenStreetMap contributors and the OpenStreetMap Foundation (https://www.openstreetmap.org), available under the Open Database License (ODbL; https://www.openstreetmap.org/copyright).


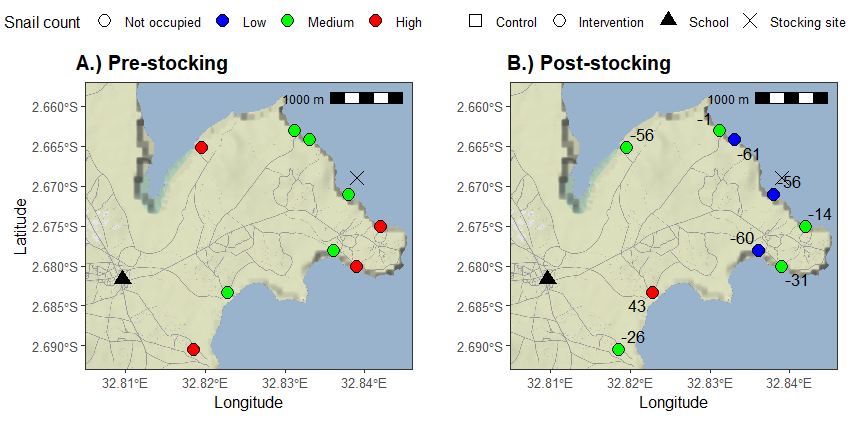


Fig C. Mean snail counts observed in the Kahumulo area by site type and period. All sites in this area were considered to be intervention sites due to their proximity to the fish intervention site (catfish stocking locations). Numbers in (B) represent change in mean count between pre- and post-stocking periods. Snail count categories were defined as: not occupied = 0, low = 0-44, medium = 44-111, and high = 111-241 snails. Base map – Stamen Terrain tiles (Stamen Design via Stadia Maps, https://maps.stamen.com), used under CC BY 4.0 (https://creativecommons.org/licenses/by/4.0/). Boundaries – Natural Earth data (public domain, https://naturalearthdata.com). Overlays – Contains information from OpenStreetMap contributors and the OpenStreetMap Foundation (https://www.openstreetmap.org), available under the Open Database License (ODbL; https://www.openstreetmap.org/copyright).
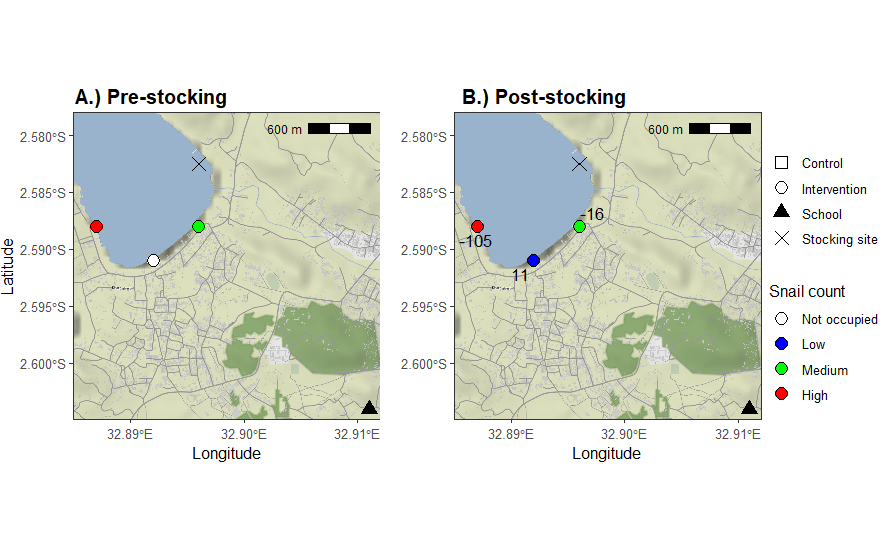


Fig D. Mean snail counts observed in the Sweya area by site type and period. All sites in this area were considered to be intervention sites due to their proximity to the fish intervention site (catfish stocking locations). Numbers in (B) represent change in mean count between pre- and post-stocking periods. Snail count categories were defined as: not occupied = 0, low = 0-44, medium = 44-111, and high = 111-241 snails. Base map – Stamen Terrain tiles (Stamen Design via Stadia Maps, https://maps.stamen.com), used under CC BY 4.0 (https://creativecommons.org/licenses/by/4.0/). Boundaries – Natural Earth data (public domain, https://naturalearthdata.com). Overlays – Contains information from OpenStreetMap contributors and the OpenStreetMap Foundation (https://www.openstreetmap.org), available under the Open Database License (ODbL; https://www.openstreetmap.org/copyright).


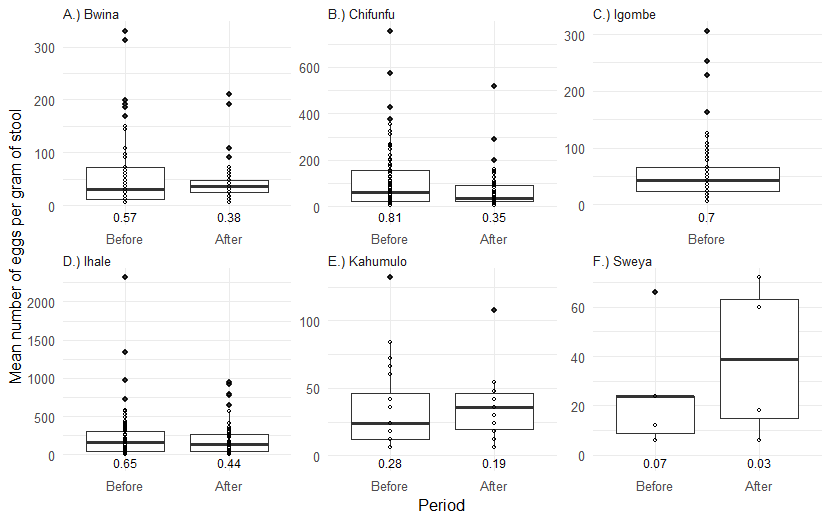


Fig E. Mean number of eggs per gram of stool for infected school-aged children (SAC) tested before and after stocking catfish based on raw observations (not considering sampling biases). Disease prevalence indicated below x-axis. The solid dots indicate outliers (1.5 times the interquartile range above the third quartile or below the first quartile). SAC at Igombe primary school were only tested in 2022 and no tests were conducted in Kayenze.

Table A. Sample size of human parasitology tests carried out by the three examiners (a, b and c) at primary schools on school-aged children (SAC) in October 2022 and November 2023. In 2022, a stool sample was obtained on consecutive days (S1 and S2). In 2023, only a single stool was tested. Not all SAC provided a second stool in 2022. SAC at Igombe primary school were only tested in 2022 and no tests were conducted in Kayenze.

|  |  | **Tests** | | **Examiners (2022)** | | **Examiners (2023)** | |
| --- | --- | --- | --- | --- | --- | --- | --- |
| **Area** | **School type** | **2022 (S1, S2)** | **2023** | **a** | **b** | **a** | **c** |
| Bwina | Control | 100 (100, 97) | 107 |  | 100 |  | 107 |
| Chifunfu | Intervention | 100 (100, 100) | 124 | 100 |  |  | 124 |
| Igombe | Control | 100 (100, 94) |  | 100 |  |  |  |
| Ihale | Control | 100 (100, 93) | 113 |  | 100 | 113 |  |
| Kahumulo | Intervention | 100 (100, 85) | 93 |  | 100 | 3 | 90 |
| Sweya | Intervention | 100 (100, 83) | 119 | 100 |  | 98 | 21 |

Table B. GLMM snail model results (fixed effects only). β is the model parameter estimate, SE is the standard error and zi is the zero-inflation component of the model (i.e. a binomial GLM used to predict probability of generating excess zeros).

|  | Count model | | |  | zi model | | |
| --- | --- | --- | --- | --- | --- | --- | --- |
| Fixed effect | **β** | **SE** | **p value** |  | **β** | **SE** | **p value** |
| Intercept (Control + Before) | 1.78 | 0.40 | < 0.001 |  | 1.08 | 0.94 | 0.25 |
| Intervention | 0.18 | 0.14 | 0.2 |  |  |  |  |
| After | 0.41 | 0.17 | 0.016 |  |  |  |  |
| Scoop-effort | 0.043 | 0.0068 | < 0.001 |  | -0.044 | 0.017 | 0.008 |
| Intervention:After | -0.85 | 0.26 | < 0.001 |  |  |  |  |

Table C. GLMM egg model results (conditional model only). β is the model parameter estimate, and SE is the standard error.

| Parameter | β | SE | p value |
| --- | --- | --- | --- |
| Intercept (Control + Before) | 1.60 | 0.09 | < 0.001 |
| Intervention | -0.27 | 0.13 | 0.038 |
| After | -0.061 | 0.17 | 0.72 |
| Intervention:After | -0.80 | 0.26 | 0.0018 |
